# Supplementary material for: Are carbon emissions trading and green financial instruments synergistic? -Comprehensive quantitative research based on content analysis
Source: PLoS One. 2024 Mar 7;19(3):e0298601. doi: 10.1371/journal.pone.0298601 (PMC10919601; doi:10.1371/journal.pone.0298601)
Supplement: S1 Data — (ZIP) [file pone.0298601.s001.zip › Data/地级市Z_F工作报告.zip!/╜¡╦╒/┴1⁄4╘╞╕█╩╨/└┤╫╘╣1⁄2╓┌║┼í╛┬φ┐╦╩2╛▌═°í┐_2009.docx]

各位代表：

现在，我代表市人民政府向大会作工作报告，请予审议，并请各位政协委员和其他列席同志提出意见。

2008年工作回顾

2008年，全市人民在中共连云港市委的领导下，以党的十七大精神为指导，全面落实科学发展观，面对宏观经济形势复杂多变的发展环境，按照中央和省决策部署，结合实际，抢抓机遇，拼搏进取，积极应对重大挑战，奋力解决突出问题，统筹推进各项工作，保持了经济平稳较快增长，社会事业全面进步，人民生活继续改善。全市实现地区生产总值750.1亿元，增长13.1%。三次产业结构调整为16.4:47.3:36.3。完成财政总收入180.6亿元、一般预算收入66.2亿元，分别增长47%、35.7%，一般预算收入占地区生产总值比重8.8%。全社会固定资产投资、社会消费品零售总额分别增长33%、24.6%。完成了市十二届人大一次会议确定的各项年度任务。

过去一年，我们主动适应宏观调控政策，积极对接国家和省推动江苏沿海开发的战略部署，狠抓第一要务，破解发展难题，主攻项目建设，加快结构调整，着力提升经济运行质量，产业经济实现又好又快发展。

新型工业化进程加快。坚持以科技进步、结构调整推进发展方式转变，促进工业经济速度质量同步提升，工业化率提高到　37.5%。依托港口、岸线、园区综合优势，加速突破临港工业，中远8万吨级浮船坞建成运营，启创铝制品、阳光晶源、益海金龙鱼一期项目竣工投产，国电风电设备制造基地开工建设；南翼灌河口船舶产业集聚地初步形成，恒成船业、五洲船舶等一批修造船项目投入生产；北翼新海石化重油深加工、金茂源生物化工项目建成投产。规模以上工业投资、增加值、销售收入分别增长37.6%、18.1%和38%。产业发展科技支撑有效增强。国家新材料高技术产业基地正式授牌，神鹰碳纤维一期工程投产，中复连众2兆瓦风电叶片下线，国家新医药产业基地成为省重点支持创建的百亿产业基地，18个项目通过国家十一五新药创制重大科技专项评审。全市新增省级以上企业技术中心3家，获批国家重点新产品5个、省高新技术产品26个，高新技术产业产值增长40.7%，占规模以上工业产值比重提高0.9个百分点。东海硅产业荣列中国百佳产业集群，太阳雨成为中国驰名商标。狠抓节能减排，完成16个污染减排项目和13家挂牌督办企业治污任务，关停小化工企业25家，单位地区生产总值能耗下降4.6%，环境质量综合指数达82.2。高度重视土地资源保护利用，大力推行节约集约用地，积极盘活存量土地，复垦开发新增耕地15000亩。建筑市场不断扩大，建筑业增加值增长8%。

现代农业规模扩张。以促进农民增收为核心，落实各项惠农政策，加快发展生态高效外向型农业。粮食总产320万吨。新增高效农业面积24.5万亩，畜禽规模化养殖比重提高6个百分点。新批农业外资项目22个，实际利用外资1.3亿美元，完成农产品出口2.9亿美元。加强农产品质量建设，新增无公害品牌78个、绿色食品品牌6个，3个标准化示范区通过国家或省验收。东海大米通过国家地理标志保护产品认定。在苏北率先创建新坝、四季田园农副产品集体商标。新增农民合作经济组织222家，市级以上农业龙头企业发展到85家。实施科技富民强县专项行动，开通农业科技人工语音服务热线，培育农业科技型企业10家、科技示范园10个，农机新机具新技术深入推广，农资农技服务网络逐步健全。

服务业发展提速。实施改造提升与增量创新发展，服务业增加值增长14.9%。港口运输列入铁道部路企直通试点，新开五定班列2条，建成西宁、侯马、淮安等物流场站，实行集装箱船舶零待时制度，完成吞吐量1亿吨、集装箱运量300万标箱，孕育百年的亿吨大港梦想成真。修编旅游发展总体规划，建成抗日山AAAA级景区，温泉度假区被评为中国十大温泉休闲基地，在中央电视台开展城市旅游形象宣传，境内外游客接待量、旅游总收入分别增长10%、12%。民航新增宁波等3条航线，客运量增长10%。中港印千万吨配煤中心建成投产，新海粮食物流中心一期工程投入使用，金港湾国际物流园建设积极推进。专业市场、连锁经营加快发展，四季农副产品批发中心建成营业，改造规范农贸市场10个，新建改建农家店400家。出台促进房地产业健康发展措施，全市商品房登记销售面积275万平方米。信息化建设加快推进，网络普及水平不断提高。金融机构人民币贷款余额比年初增加103.5亿元，有力支持了地方发展。

过去一年，我们以新一轮思想解放为动力，加快推进体制改革和机制创新，努力拓展对外开放的深度广度，区域经济发展彰显蓬勃的生机活力。

各项改革向纵深推进。顺利调整市区行政区划，稳步推进灌云乡镇机构改革试点，东海乡镇纪检监察资源重组改革经验在全国推广。事业单位公开招聘制度进一步规范完善，市属生产经营型事业单位改制转企圆满完成。投融资体制改革实现新突破，组建新海连集团，完成新苏港公司增资扩股，成功发行连云发展集团10亿元企业债券。金融创新取得进展，组建完成市县邮政储蓄银行，东海村镇银行挂牌营业，灌南、东海农村小额贷款公司完成组建，灌云农村资金互助社开始运作，金融服务多元化步伐加快，农业保险范围逐步扩大。公共财政框架体系不断完善，税收属地征管运行良好，非税收入改革顺利实施。在全省率先实行按面积分配城市道路绿化养护费改革。促进民营经济发展壮大，新增私营企业5133家、个体工商户注册数2.7万户、从业人员8.6万人，12家企业入选省首批科技型中小企业，民营经济增加值占地区生产总值51%，成为吸纳就业、推动发展的重要增长点。

对外开放继续扩大。充分利用多重机遇，创新思路办法，加快汇聚要素资源，为经济发展注入动力。深化针对性研究，强化招商引资工作，积极拓展与海内外大企业合资合作，成功承办第四届苏北投资贸易洽谈会、全国台企联常务理事会，利用苏台经贸洽谈会等各类招商平台广泛招引外来资源，实现注册外资实际到账9.35亿美元，内联客方到位资金329亿元。积极应对国际市场变化，调整优化出口产品结构，保持对外贸易稳健发展，完成出口22.9亿美元，增长24.1%，总额继续居苏北之首。加快提升园区载体功能，市经济技术开发区获批省国际服务外包示范区，在国家级开发区排名较上年上升1位，东海开发区硅材料工业园等3个园区被评为省级特色园区，连云港化学工业园通过ISO双体系认证。市临港产业区基础设施配套逐步完善，两翼园区建设全面推进，沿海临港产业园区新开工重点项目占全市60%。开展专项整治，有效规范出国劳务市场。外事服务水平进一步提升。加快优势产业企业走出去步伐，新增境外投资企业7家。

过去一年，我们注重增创发展优势，推动提升战略地位，加快基础设施建设，完善城市功能，改善农村发展条件，全市跨越发展的支撑能力显著提高。

港城发展正在进入国家战略层面。国务院总理办公会研究江苏沿海开发，把连云港作为重中之重强调，国家发改委牵头组织调研组来连调研，通过我们主动对接、积极争取，我市战略区位、发展思路进一步得到提升。国务院出台的长三角地区改革开放和经济社会发展指导意见明确要求加快连云港发展，形成新的经济增长点，带动江苏沿海、东陇海沿线发展。正在编制的江苏沿海地区区域发展规划将从国家战略高度，确认连云港在带动区域经济协调发展中的重要地位，为我们拓展发展空间、增创发展优势提供了历史性机遇。

重大基础设施建设取得明显成效。投入53亿元实施主体港区深水航道、码头、疏港道路等重点工程，15万吨级航道建成通航，庙三突堤5个集装箱泊位、焦炭专业化泊位基本建成，30万吨级矿石码头具备靠泊条件，南疏港道路二期工程主体建成，东疏港道路快速推进，铁路1050站场改造竣工验收，港口综合通过能力增加1600万吨。连临高速、310国道、242省道、疏港航道、盐灌船闸等重点交通工程加快推进。实施通榆河北延、大浦河调尾、新沭河治理等大型水利工程，完成新沂河整治。加大电力、通讯等设施投入，220千伏台北、金庄输变电工程等一批电网建设项目建成运转，信息网络基础设施进一步完善。

国际性海滨城市功能不断增强。全面拉开三极互动的特大城市框架，完成城建投入80亿元，中心城市建成区面积扩大到95平方公里，城市化水平达到42%。新一轮城市总体规划完成修编，港口总体规划获部省联合批复，城市规划展示馆建成开放。东部城区建设加快推进，实施27个重要地块建设改造，海滨新城启动区有序进展，完成海域匡围4500亩，抛填形成陆域、回填场地6000亩，东方国际商务中心主体工程完工，北崮山大道、西墅路等骨干道路实现通车，海棠路立交、中云客运站等重点工程加快建设。新海城区东河、凤凰、孔望山、花果山片区开发有效推进，花果山大道建成通车，科教创业园区初现形象。新建改建城市主次干道30条、自来水干线45公里，增加管道天然气用户8000户、集中供热50万平方米，完成人防工程14万平方米，开工建设大圣湖应急供水安全工程。推进绿色连云港建设，加强林木管护，城市绿化覆盖率达40.5%。优先发展城市公共交通，新建新浦汽车客运总站，新增和优化一批公交线路。加大环境卫生整治和基础投入，城市污水处理污泥焚烧设施建成使用，生活污水集中处理率达80%。

新农村建设取得新进展。坚持以人为本，突出特色优势，加快推进县乡规划建设。修编四县县城总体规划，实现村镇规划全覆盖。新农村规划居住示范点建设积极推进，村级综合服务中心建成率100%，赣榆县海头镇成为国家发展改革试点镇。建成农村公路1385公里，改造危桥45座，新建城乡客运一体化候车亭216个，行政村客运班车通达率99.2%。农业综合开发有效推进，完成中低产田改造15万亩，治理水土流失50.8平方公里。综合整治农村环境，全面启动农村饮用水安全工程，完成30万人改水任务，新增农村无害化卫生厕所3万个，发展户用沼气1.5万户，疏浚县乡河道208条，累计创建市级环境优美乡镇36个、省级生态村8个，农村面貌不断改观。

过去一年，我们坚持统筹经济社会发展，开展文明城市创建，加快科教文卫等各项社会事业建设，推进公共服务均等化，力促社会文明和谐。

科技教育全面进步。加强创新型城市建设，加快释放第一生产力。成功引进中国科学院先进能源动力技术研发中心，建成市高新技术产业区科技创业园和科技成果转化中心，国家级博士后工作站发展到7家，新上省级以上科技计划项目124项、国际科技合作计划8项。专利申请1000件、授权330件，获中国国际发明展览会金奖6项。推进教育现代化建设，提升基础教育水平，促进教育均衡协调发展。对所有中小学校舍进行抗震安全排查，改扩建校舍60万平方米，小学、初中入学率基本达到100%，高中段毛入学率92.1%。创建省三星级以上中等职业学校4所，新增省级示范专业14个，中等职业学校毕业生就业率96.6%。地方高等教育加快发展，连云港职业技术学院迁入新校址，师专新校区一期工程建成，工贸高职学校主体封顶。落实义务教育免费政策和贫困生资助制度，减免和发放资金3.5亿元，惠及学生117万人次。新设国家级引进国外智力示范基地，获批省高层次创新创业人才引进计划7项，引进本科以上学历人才3600人。

社会事业协调发展。加快建设覆盖城乡的基本医疗卫生制度，城市社区卫生服务人口覆盖率90%，农村卫生服务体系健全率98%，新型农村合作医疗参合率和农民住院医疗费用实际报销比例高于省平均水平，基本建立城市居民15分钟健康服务圈。开展惠民医疗，免费为1000余名白内障患者实施复明手术。完善公共卫生服务体系，市第一人民医院、中医院晋升三级甲等医院，二院东院区建成启用，海港医院并入市一院东方医院，疾病预防控制水平不断提升，法定报告传染病发病率低于省平均水平。加强人口和计划生育管理服务，人口自然增长率控制在6.9‰以内，计划生育率90%以上。加快公共文化服务体系建设，建成村级文化室844个、农家书屋488个，乡镇文化站全部达标。加大文物和非物质文化遗产保护力度，繁荣文艺创作，57件文艺和广播电视作品获省以上奖项。有线电视入户率达61.7%。市体育中心体育馆主体工程完工，乡村两级全民健身设施网络基本形成。广泛开展城乡文化体育活动，成功承办中国乒乓球俱乐部甲A联赛等重要赛事，我市运动员获得残奥会奖牌数实现历史性突破。妇女儿童事业加快发展，市妇女活动中心完成主体工程。加强国民经济统计分析和重点建设项目跟踪审计工作。侨务、气象、档案、地方志、民族宗教、防震减灾、老区开发等工作取得新成绩，红十字、关心下一代、老龄事业、光彩事业、慈善事业再上新台阶。

精神文明建设和社会管理取得新成效。全民动员、全社会参与，全面掀起文明城市创建热潮，通过加强软硬件建设，推进长效管理，市民文明素质得到提升，市区和东海县顺利通过省级文明城市创建验收。建成雷锋车城雕和雷锋车事迹陈列馆。成功开展了八千里路走连霍大型采访活动，《苍梧晚报》荣获全国未成年人思想道德建设工作先进集体。8家商品交易市场成为省四星级以上文明诚信市场。全力以赴支援地震灾区抗震救灾，捐款捐物1.1亿元，完成对口援建什地镇灾后重建年度任务。开展安全生产执法年活动，重点领域专项整治工作取得成效。公共突发事件应急预案逐步完善，应急指挥平台投入使用。完成处置浒苔灾害支援奥运任务。进一步加强国防建设、民兵预备役和优抚安置工作。推进法治连云港、平安连云港建设，开展领导干部大接访活动，信访基层基础工作得到加强，强化青少年违法犯罪预防工作，建立100个乡镇、街道法律援助站，完善城市治安设防工作，科技强警示范城市建设通过公安部、科技部考核验收，群众安全感进一步增强。

过去一年，我们坚持以人为本、富民优先，着力解决群众最直接、最现实的利益问题，推动就业惠民、创业富民、社保安民，城乡居民收入较快增长，民生不断改善，群众得到实惠。

就业规模进一步扩大。促进发展经济与增加就业良性互动，推进统筹城乡就业，新增就业人员5.9万人，城镇登记失业率3%。积极扶持创业带就业，全市发放小额担保贷款5246万元，帮助3173人就业。加强职业技能培训，开展充分就业社区、充分转移乡镇和行政村创建活动，完成各类职业技能培训24.3万人次，新增农村劳动力转移8.1万人。多渠道增加就业岗位，落实就业优惠政策，健全就业困难群体长效帮扶机制，开发公益性岗位577个，帮助下岗失业人员再就业2.4万人，就业困难人员就业5293人，零就业家庭动态清零。全市城镇居民人均可支配收入15255元，农民人均纯收入5454元，分别增长15.1%、13%。

社会保障体系进一步健全。不断扩大社会保险覆盖面，执行基本养老保险新政策，将城镇企业职工养老保险延伸到城乡各类企业，全市基本养老保险参保人数达35万人。提高企业退休人员养老待遇，四县全部建立新型农村养老保险制度，参保人数达14.9万人。做好被征地农民社会保障，做到先保后征，确保失地农民失地不失业，基本生活长期有保障。建立建筑业农民工参加工伤保险制度。完善基本医疗保险政策，提高参保人员医保待遇。

发展成果共享面进一步扩展。建立财政投入向公共服务领域倾斜的支出保障制度，进一步加大科教文卫、社会保障等领域保障力度，城乡居民享有的公共服务明显增多。经济适用房竣工17.2万平方米，为1617户低收入家庭提供廉租房保障。建设便民道路65条，整治人行道板5万平方米。归集城镇职工住房公积金11.1亿元。流浪未成年人救助保护中心建成使用，城乡社会救助体系逐步健全。市社会福利中心启动建设，落实老年福利养老机构床位补贴，农村五保户集中供养率提高到65.7%。实施脱贫攻坚工程，实现10万农村贫困人口增收脱贫。影响群众生活的突出问题逐步得到解决，推行阳光拆迁，提升食品药品安全监督和公共卫生应急处置能力，有效应对婴幼儿奶粉事件，开展乳制品专项整治，药品生产经营环节监督率100%，零售企业远程监控率56.3%，居民消费价格涨幅4.8%，低于省平均涨幅。

过去一年，我们坚持执政为民、依法行政，发展社会主义民主，提高行政执行力，推进为民、务实、高效、廉洁政府建设。

改进工作作风，提高服务效能。创新政府服务管理方式，加快电子政务建设，推行网上办公，健全政务公开制度，在政府门户网站公开政府工作报告目标任务推进落实情况，接受公众监督，强化重大事项督查推进，保证行政效能提高和一批重点工作落实。进一步精简会议文件，严格控制评比达标表彰活动，全市性会议比上年减少60.7%。深化行政审批制度改革，调整取消行政审批事项30项，取消和停止征收行政事业性收费、政府性基金61项，初步建立覆盖全市的四级便民服务网络体系。

坚持从严治政，推进依法行政。自觉接受市人大的法律监督、工作监督和市政协的民主监督，坚持重大事项向市人大常委会报告、向市政协通报，注重发挥各民主党派、工商联、人民团体和无党派人士的参政议政作用，共办理人大代表建议163件，政协委员提案397件，办复率100%。推行重要政策措施公示制度，提高行政决策民主性。强化行政执法监督，规范自由裁量权。认真贯彻惩治和预防腐败体系工作规划，加强反腐倡廉工作，努力树立新一届政府良好形象。

各位代表，回顾过去一年工作，在宏观经济形势急剧变化、发展压力不断加大、各种困难和矛盾日益凸显的情况下，取得较好发展成绩，我们深感来之不易。这是中共连云港市委正确领导的结果，是市人大、市政协和各位代表、各位委员监督支持的结果，是全市人民和衷共济、团结奋斗、共克时艰的结果。在此，我代表连云港市人民政府，向辛勤工作在各条战线的全市广大干部群众致以崇高的敬意！向大力支持政府工作的人大代表、政协委员、各民主党派、工商联、人民团体和各界人士，向积极参与连云港建设的在连部省属单位、驻军部队、武警官兵、公安干警，向关心连云港发展的海内外朋友，表示衷心的感谢！

在充分肯定成绩的同时，我们也清醒认识到，我市经济社会发展还面临不少困难和挑战，政府工作中还存在一些矛盾和问题。一是受世界金融危机蔓延波及，我市港口运输、产业发展、招商引资、对外贸易外部环境趋紧。二是经济运行中一些突出问题和深层次矛盾依然存在，重大项目支撑拉动不够突出，企业抵御市场风险能力还不强，随着工业化步伐加快，节能减排任务更加艰巨。三是关系群众切身利益的就业、住房、医疗、教育等问题有待更好解决，城乡社会保障水平需要继续提高，社会建设和文化发展等方面基础还较薄弱。四是政府自身建设需要进一步加强，社会管理和公共服务能力有待进一步提升。对这些问题，我们将高度重视、认真解决。

2009年主要工作任务

2009年是新中国成立60周年的喜庆之年，是我市跻身国家发展战略、在新的起点上加快开发建设的起始之年，也是实现十一五发展目标的关键一年。做好今年工作，意义十分重大。

综观国内外形势，发展的外部环境更加严峻，各种风险和挑战前所未有。我们突出面临着世界经济环境恶化导致的需求性调整压力，国内经济多年快速增长带来的周期性波动压力，人口资源环境约束形成的结构性压力，这些因素对经济的挑战不是短期内能够解决的。受其影响，我市经济下行压力进一步加大，企业生产经营困难，财税增长放缓，就业压力明显增加，农民持续增收难度加大。但是，困难中蕴藏希望，挑战中孕育机遇。我们在看到困难与挑战的同时，更要看到有利条件和积极因素。我们的有利条件是党的十七届三中全会为农村改革发展注入新的动力，作为农业大市，只要我们科学运作、敢闯敢试，坚定不移地以改革创新引领发展，就能激发更多活力、释放更多资源、调动更多积极因素。我们的有利条件是国家新一轮扩大内需政策措施，给我市大港口、大交通、大产业发展带来了现实机遇，只要我们迅速行动、有效对接，适时实施一批重大基础设施、产业和民生项目，就能化危机为转机、转压力为动力、变被动为主动。我们的有利条件是近年来一体两翼组合大港、一心三极海滨城市、一纵一横T型产业走廊建设快速推进，全市跨越发展基础日趋坚实，特别是连云港将跻身国家发展战略，为新一轮发展带来了千载难逢的历史机遇，只要我们用好机遇、乘势而上，在更高层次上吸纳更多优质要素，就能把政策优势转变为经济优势、资源优势转变为产业优势、后发优势转变为发展优势。

我们欣喜地看到，尽管当前宏观经济形势风云变幻，但全市工业化城市化进程提速的趋势没有变，经济社会发展步入快车道的基本面没有变，480万人民奋发进取、建设美好家园的昂扬斗志没有变，连云港的发展也从来没有象今天这样充满活力，这是我们加快推进跨越发展的重要基础和根本动力。我们坚信，只要全市上下振奋精神，团结一致，迎难而上，埋头苦干，就一定能够战胜任何困难和挑战，把重振连云港的事业继续推向前进！

根据中央、省经济工作会议和市委十届七次全会部署，今年政府工作总的要求是：全面贯彻党的十七大和十七届三中全会精神，以邓小平理论和三个代表重要思想为指导，深入贯彻落实科学发展观，突出力保强劲增势、提升跨越后劲年度工作主题，以扩内需、促增长、调结构、提质量、重民生为主线，进一步解放思想、抢抓机遇，深化改革、扩大开放，积极应对宏观经济形势变化，统筹推进经济社会各项工作，确保经济快速健康发展，确保重点项目强力推进，确保民生逐步改善，确保社会和谐稳定，创造科学发展、跨越发展新业绩。主要预期目标为：地区生产总值增长13%，财政一般预算收入增长18%，全社会固定资产投资800亿元，居民消费价格涨幅保持相对稳定，节能减排完成省下达任务。

围绕实现上述目标和要求，新一年政府工作的重点是放大机遇优势，增加有效投入，调整产业结构，推动创业创新，加快海滨城市建设，推进农村改革发展，保障民生改善。具体抓好六个方面工作：

一、把握重大机遇，重抓增创跨越发展新优势

我们将抢抓扩大内需和进入国家战略的机遇，按照江苏沿海开发战略部署，顺应财政货币宏观政策重大调整，加快实施一批关系全局的重大基础设施和产业项目，为推进科学发展、跨越发展提供新的支撑。

以加快港口建设为龙头，大力实施重大基础设施工程，增强发展承载功能。在亿吨大港的新起点上，开工建设30万吨级深水航道工程，全面建成30万吨级矿石码头，基本建成氧化铝、散化肥等专业化泊位，加快建设旗台防波堤。推进疏港航道和内河港工程，建成南疏港通道，完成东疏港道路主体工程。启动徐圩港区起步工程和10万吨级航道建设，积极推进灌河港区和赣榆港区开发。加快实施通榆河北延、新沭河整治、大浦河调尾等重点水利工程，推进海堤大中型挡潮闸建设。进一步完善便捷通畅交通网，完成连临高速公路路基工程，加快推进310国道东海段、242省道、204国道赣榆段、324省道灌云段、226省道连云港北延段、灌南新港大道建设。建成盐灌船闸，实现海河联运。扩建白塔埠机场国际候机楼。开工建设连盐（淮）铁路。加大电网改造力度，建成500千伏伊芦、220千伏申城、龙河等一批输变电设施。积极推进3G通信网络工程建设。

以兴办大型临港产业项目为突破口，加快推进沿海开发，努力培育新的增长点。把发展大型临港产业作为沿海开发重中之重，加快推进石化、钢铁、造船、能源等重大产业项目建设。积极培育石化产业，启动实施新海石化二期项目。争取建设南钢精品钢铁基地一期工程，推进胜恒钢制品项目。加快灌河口船舶产业集聚地、中远修造船基地建设，完工造船总量100万载重吨。建设新海发电公司1000兆瓦超超临界火电机组，推进田湾核电新上两台百万千瓦机组扩建工程和国电集团火力发电项目。加快实施中彩多晶硅、30万吨氯碱、华磁商用电器等一批大项目。坚持以规划引领开发，抓好沿海产业、港区园区、生态系统等规划编制。全面推进徐圩片区开发，完善柘汪、板桥、燕尾、堆沟等临港产业园区功能，为沿海开发搭建良好产业平台。

以推进一批重点事项前期工作为着力点，提升跨越发展后劲，争取新一轮开发主动权。有效对接跻身国家战略和扩大内需政策，从战略层面策划、编制一批事关长远的重点事项，加快推进前期工作，促进一批多年来努力争取的重大产业项目和发展事项尽快报批、早日开工。重点开展1000万吨炼油和100万吨乙烯、原油储备基地、煤气化新能源、LNG等重大产业项目前期工作。加快启动区域性大型机场选址，推进连云港至上海、南京、青岛高铁，西安至徐州客运专线东延，北疏港道路、盐河、排淡河、蔷薇河和拦门沙治理等重点交通水利设施项目。积极争取保税港区，认真开展国家区域协调发展示范区等一批政策课题研究，为项目报批实施做好准备。

二、推进结构调整，重抓发展方式转变新突破

我们将把促进转变发展方式、增强经济竞争力，作为应对严峻挑战、保持经济增长的主攻方向，立足地方实际，调整优化经济结构，构筑现代产业体系，提高经济增长质量和效益。

加快发展先进制造业。以推进主导产业规模化、新兴产业高端化、传统产业名牌化为取向，努力打造沿海新型先进制造业基地。大力发展高新技术产业，实施新一轮双倍增计划，推进国家新材料高技术产业基地建设，促进新医药产业做大做强，加快传统产业高新化改造，打造江苏新的高新技术产业集聚区，完成高新技术产业产值230亿元。着力推进产业集群发展，围绕加快培育千亿产业和百亿企业，积极构建具有较强竞争优势的硅材料、碳纤维、风电装备、盐化工等特色产业链，打造一批骨干企业，销售收入过10亿元企业发展到30家。以信息化带动工业化，培育信息化示范企业10家，三新产业和现代装备制造骨干企业的信息化集成技术应用覆盖率达50%。深入实施专利、标准和品牌战略，提高百万元地区生产总值专利授权数。继续推进质量兴市，强化产品质量监管，加大名牌培育力度，积极创建中国驰名商标。进一步优化投资结构，引导工业投资更多投向高端制造、创新研发、节能环保等重点领域，加大政策扶持，改进协调服务，加快中复神鹰碳纤维、中材高新硅材料、远洋流体装备二期等100个新增长点和100项重点工业项目建设，完成工业投资550亿元，工业增加值增长15.5%。建筑业增加值增长10%。

大力发展现代服务业。以扩总量、调结构、增效益为目标，促进服务业发展提速、比重提高、层次提升，服务业增加值增长15%。加快现代物流、金融保险、研发设计、中介服务等生产性服务业发展，推进国际商务中心、省级国际服务外包示范区、连云港软件园等现代服务业集聚区建设，抓好万邦物流园、金港湾国际物流园等一批服务业基础设施重点项目。积极构筑现代物流体系，加快无水港建设，完善口岸信息平台功能，加大揽货力度，增辟航线12条，完成港口吞吐量1.1亿吨，集装箱运量300万标箱，力争空运客流量30万人次。加快发展商贸流通，推进国贸中心、苏宁大厦、国际商城等一批重点项目建设，加强特色商业街区建设，大力发展连锁经营、物流配送，拓展农村、社区连锁销售终端网络，农家店发展到1700家，建设区域性农产品物流中心，社会消费品零售总额增长16%。实施旅游资源深度开发和保护，加强服务能力建设，加快产业提档升级，境内外游客接待量、旅游总收入分别增长6%和10%。大力培育文化产业，积极发展创意、动漫等新型业态，文化产业增加值增长15%。改进银企合作模式，加强对中小企业和三农金融服务，金融机构人民币贷款余额增长18%，增幅高于全省和苏北平均水平。

积极推进科技创新。加快自主创新和引进消化吸收再创新，发展提升优势产业，培育壮大新兴产业。强化企业自主创新主体地位，引导和鼓励企业增加研发投入。不断提高全社会研究与试验发展经费支出占地区生产总值比重，科技进步贡献率达到37%。加快重大科技创新平台建设，完成中科院先进能源动力研发中心一期工程，江苏海洋资源开发研究院形成科研能力，建设国家硅产品质量监督检验中心。推进产学研更加紧密结合，促进科技成果产业化应用。培育高新技术产品40个，高新技术企业20家。加快创新载体建设，新增科技孵化器面积2.5万平方米。

认真抓好节能减排。推行投资项目节能评估审查，开展清洁生产，发展循环经济，推进资源综合利用。实施20项重点节能改造项目，加快淘汰集中供热范围内小锅炉。开发农村能源综合利用技术。实行全民节能行动，倡导绿色消费。单位地区生产总值能耗下降完成省下达任务。加强对重要水源地、河流沿线排污口监管，启动建设新沂河尾水排放通道，加快实施烟气脱硫等重点治污项目，整治机动车尾气排放，开展排污权有偿转让试点。全市化学需氧量削减2.2%，二氧化硫排放量减少3.1%。

三、坚持城乡统筹，重抓开创农村发展新局面

我们将认真贯彻十七届三中全会精神，以致富农民为核心，以结构调整为主线，以改革创新为动力，实施新农村建设六大行动计划，加快城乡一体化发展，繁荣农村经济，增加农民收入，改善农村面貌。

大力发展生态高效外向农业。调整优化产业、品种结构和空间布局，建设长三角地区优质农产品基地。突出设施农业和规模养殖，抓好精深加工和市场流通，强化质量安全和品牌建设，进一步加快农业产业化、园区化、外向化、标准化发展，新增高效农业20万亩，大力培育规模龙头企业，新办农产品加工企业80家，规模养殖比重提高5个百分点。积极发展花卉苗木和休闲农业，建成全省重要的鲜切花生产基地。加大农业招商力度，利用外资增长10%，做大做强20个出口农产品生产基地和20个骨干出口企业，农产品出口增长15%以上。建立完善农业标准、检验检测、执法监督体系，争取定点建设国家级农产品质量安全监测中心。

着力改善农村生产生活条件。把致富农民和壮大村级集体经济作为改善农村发展条件的重要途径。建立促进农民增收长效机制，培育农民创业主体，扶持农民专业合作组织和农村经纪人，加强农民劳动技能培训，培训农民20万人，新增农村劳动力输出5万人，全市农村劳动力培训后转移就业率60%，农民专业合作和土地股份合作组织达800家，培育发展农村经纪人2000人。健全农技服务体系，建立新型乡镇畜牧兽医体系和专业队伍。做大做强村级集体经济，增加集体收入过20万元村70个。加快改善农村发展物质条件和公共服务，扎实办好新一轮实事，推进生产保障性基础设施建设，加强农业综合开发，搞好灌区改造、水库除险加固、小型泵站更新，改造中低产田11万亩，新增户用沼气1.2万户、水稻插秧机1000台，建成农村公路500公里，完成166条河道疏浚和2009个河塘整治。继续实施农村饮用水安全工程，完成51万人改水任务。推进城乡一体化规划编制，加快县城开发建设，打造一批特色乡镇。

认真落实支农强农惠农政策。探索稳定完善农村基本经营制度新途径和农村土地流转举措，进一步放开搞活农村经济，建立流转土地储备库，增加流转面积10万亩。兑现国家提高粮食最低收购价、增加农业补贴政策。建立健全财政支农支出稳定增长机制，积极调整财政支出、固定资产投资、信贷投放结构，保证各级财政农业投入增长高于经常性收入增长，土地出让金、耕地占用税、新增建设用地有偿使用费等土地收益用于农业农村部分足额落实到位。今年市级财政新增投入3000万元以上，加大对三农支持力度。扩大政策性农业保险覆盖面。加快实施脱贫攻坚工程，实现10万贫困人口脱贫。

四、深化改革开放，重抓增强崛起振兴新动力

我们将坚持把改革创新作为一切工作的活力源泉，进一步扩大开放，加快集聚各类要素资源，为跨越发展注入活力、拓展空间、增强后劲。

加大重点领域和关键环节改革力度。推进投融资体制改革，利用国家实行适度宽松货币政策有利时机，加快投融资平台建设与运作方式创新，扩大投融资规模。大力推进企业上市工作，推进上市公司再融资，争取发行港口集团企业债券。整合国有资本运营主体，进一步完善国有资产管理体制。加强信用和担保体系建设，扩大担保贷款规模，落实微小企业牵手成长助贷工程，切实解决中小企业融资难题。建立完善农村金融组织体系，发展小额信贷和微型金融服务，四县全部组建农村合作银行，县区全部建有小额贷款公司。全面实行增值税转型和成品油价格税费改革。积极推进医疗卫生体制改革，建立健全医疗卫生服务新体系。开展涉企行政事业性收费专项清理，减轻企业负担，让企业休养生息。加快推进乡镇机构改革。

提升新形势下对外开放水平。抓住世界金融危机冲击下新一轮产业、资本、技术、人才资源重新整合的契机，把扩大开放与应对金融危机、调整优化产业结构、增强区域服务功能紧密结合，构筑开放型经济新格局。强化多重叠加优势宣传推介，创新招商引资方式，深化产业招商、专业招商、以商引商。推进三新产业、石化钢铁、现代服务业、高效农业和城市建设、科技教育等全方位、多领域的大招商、招大商。研究差别化、具体化扶持政策，实施领导干部公务出国（境）挂钩重点招商目标地制度，以160个重大项目为抓手，务实搞好日韩、港台、长三角地区等12个重点招商活动。改善投资服务，促进在建项目投产达效。注册外资实际到账10亿美元，引进内联客方到位资金350亿元。以国家级省级开发区为重点，完善配套服务，推进南北共建，提升开发开放层次，提高投入产出效益。促进对外贸易健康发展，落实扶持政策，开拓新兴市场，优化出口产品结构，扩大机电和高新技术产品、农产品出口，推进加工贸易转型升级，外贸出口增长10%。完成出口加工区二期工程封关运作。提升外经合作层次，支持有条件企业开展国际化经营。发挥外事、台办、侨务等领域的外联优势，扩大对外经济交往与合作。

推进新一轮全民创业。认真研究支持群众创业的方式，完善劳动者创业帮扶政策体系，加强创业载体和服务平台建设，设立创业投资和风险投资资金，建设市科技创业服务中心，积极探索创业保险、小额担保和农村住房抵押贷款，为创业提供支持。加大公共财政支持力度，健全创业教育培训和政府资助资金使用制度，提高劳动者创业能力。拓展创业领域，推进公平准入，形成低门槛、多层次、宽领域的创业格局。今年新增私营企业5500家、个体工商户3万户以上，带动就业8万人。加强创业先进典型的宣传引导，积极推广东海农民工凤还巢返乡创业经验，努力形成鼓励创业、宽容失败的文化氛围，让港城大地创业的激情热潮澎湃、潜能急剧迸发、财富源泉涌流。

五、打造海滨特色，重抓城市品质新提升

我们将以国际水准规划体系为引领，强化基础设施和配套建设，完善城市功能，提升城市品质，加快建设令人自豪和向往的国际性海滨城市。

加速刷新城市形象。围绕彰显滨海特色，中央商务区加快实施在海一方商务休闲区、山海景观轴工程，推进北固山森林公园建设，兴建金海国际商务大厦、金港湾商务大厦等一批标志性建筑，开工实施平山广场、连云大厦等一批重要节点工程。海滨新区重点加快填海造陆和基础设施建设步伐，推进东方国际商务广场等一批项目建设。精心打造花果山大道山海之轴。加快新海城区东河、孔望山、凤凰和花果山片区建设，成片实施市政公用、环境景观、商住办公等城市建设项目，推进科苑路周边建设大厦等13项工程建设。积极开展旧城改造，完成60万平方米改造任务，尽快让住在低矮简陋平房的市民搬进宽敞舒适楼房。

加快完善配套功能。重点加大公用基础设施投入，增强对产业和人口发展承载能力。进一步畅通道路交通，着力加快环路建设、完善城区路网、提高通达能力。东部城区实施环山路，打通大港路，延伸海滨大道，建成海棠路立交桥。新海城区新建、续建科苑北路、振海路等主次干道20条，启动建设三环路网。保障供水供气供热安全稳定，完成大圣湖应急供水安全工程，新建改建城区自来水管道30公里，推进区域供水和城乡联网供水，实施市第三水厂、茅口水厂水质深度处理工艺改造。扩大西气东输气源供应量，新建燃气管道30公里，增加管道天然气用户7000户。推广城市集中供热，完成市区和工业园区供热主干网建设，新增集中供热面积50万平方米。加强城镇污水处理，抓好污水处理厂和管网配套建设，新建城区污水管道25公里，基本贯通新海城区污水截流主管网，建成东海尾水通道，市区主要河流水质明显改善。

加大城市管理力度。从今年开始用两年时间在全市基本建立数字化城市管理新模式，提升城市管理精细化水平。加快便民工程建设，修建便民道路30条，新装小街巷路灯800盏，整治人行道板5万平方米，新建生活垃圾填埋场等一批环卫设施，创建一批市容示范工程，实现市区卫生保洁全覆盖。加大违章建筑查处、违规广告清理和三乱小广告整治力度，有序推进市区夜景亮化、美化建设。坚持公共交通优先发展，加快公交枢纽站和停车场点建设，努力解决交通拥堵和停车难问题。鼓励利用地下人防空间开办公共停车场，完成人防工程10万平方米。

加强生态环境建设和资源节约利用。青山、碧海、蓝天是连云港引以自豪的生态资源，是广大人民共同福祉。我们一定以对子孙后代、对历史负责的态度，坚定不移地坚持环保优先，保护资源环境，建设生态文明。今年空气质量良好天数保持90%以上。继续推进四城同创，加大国家园林城市和卫生城市创建力度，争取通过国家环保模范城验收。严格保护集中式饮用水源地，确保让群众喝上干净水、放心水。加强危险废弃物管理与处置，抓好施工扬尘、餐饮业油烟排放整治，解决噪声扰民等问题。推广生态种养模式，加大森林保护和城乡绿化力度，实施30项城市绿化重点工程，新增城市绿地220公顷，城市绿化覆盖率提高0.5个百分点。推进生态修复，加强海洋管理和环境保护。实行最严格的土地管理制度，落实耕地保护责任制，加强土地资源复垦开发，健全依法用地长效机制，提高土地节约集约利用水平。

六、加强社会建设，重抓保障群众得到新实惠

我们将突出以人为本，致力推进基本公共服务均等化，提高公共产品和公共服务供给能力，坚持统筹经济与社会发展，更加重视民生改善，使民生问题得到制度化安排、长效化推进、项目化落实、系统化解决，努力让老百姓分享到更多发展成果，感受到更多公平正义，体验到更多和谐幸福。今年优化公共财政支出结构，在社会保障与就业、科技创新与创业、改善办学条件、化解教育债务、医疗卫生等方面新增财政投入全部超过1000万元。

加强就业和社会保障，致力富民惠民。有效应对经济形势对就业的不利影响，把稳定就业放在突出位置，落实更加积极的就业政策，努力实现充分就业。抓好城乡统筹就业，推动城镇就业再就业政策向农村延伸，带动劳动力转移工作整体推进。强化对劳动密集型企业帮扶，稳定和增加就业岗位。加大对低收入家庭扶持力度，做好就业困难群体援助工作，重点帮扶零就业家庭、高校毕业生就业。城镇登记失业率低于4%。城镇居民人均可支配收入、农民人均纯收入增长10%。加快完善覆盖城乡的社会保障体系，突出基本养老、基本医疗、最低生活保障等重点，逐步提高统筹层次和保障水平，城乡基本社会保险覆盖率达84.9%。继续提高企业退休人员养老金水平。全面建立新型农村社会养老保险制度，完善提高农民生活最低保障制度。解决残疾人在就业和社会保障等方面实际困难。加大保障性住房建设力度，解决城市低收入群众住房难问题，新增经济适用住房22万平方米，廉租住房租赁补贴实现应保尽保。继续提供人才安置房100套。逐步改善农民工和外来人员住房条件。加快建立新型社会救助体系，健全城乡困难群众救助标准自然增长机制，扩大救助范围。推进社会福利社会化进程，加快市社会福利中心建设，提升养老福利服务机构专业化服务管理水平。加快推进市老年大学新址改造，落实老年人优待政策。

强化教育和人才支撑，推动文化繁荣。坚持教育优先发展，加快推进教育基本现代化。促进城乡基础教育均衡发展，学前教育毛入学率90%以上，小学、初中入学率达99.98%、99.7%，外来务工子女义务教育入学率99%，高中段教育毛入学率93%。实施农村留守儿童食宿条件改善和合格幼儿园建设工程，进一步改善农村学校办学条件。在义务教育学校实施绩效工资，对离退休人员发放生活补贴。强化职业教育实验、实训工作，启动建设市高技能人才公共实训鉴定基地，做大做强市中等职教集团，重点发展农村职业教育。加快高等教育发展，推进淮海工学院等院校提升办学层次，积极争取国内外重点院校来连合作办学，加快人才培养，继续实施人才引进工程。进一步完善义务教育免费政策和经费保障机制，保障经济困难家庭子女、留守儿童平等就学、完成学业。积极推动义务教育向学前教育和高中阶段延伸。加快科教创业园区建设，进区院校、研发机构基本建成。大力发展特色文化，积极发展公益性文化事业，推进文化产业发展，加强文化遗产保护，开工建设影视文化城、水晶博物馆，村级文化室达标覆盖率100%。完成市区数字电视整体转换，有线电视入户率达70%。加强城乡公共体育设施建设，广泛开展全民健身活动，建成市体育中心体育馆。

推进精神文明建设，协调发展各项社会事业。巩固提高文明城市创建成果，启动全国文明城市创建工作，弘扬以改革创新为核心的时代精神，广泛开展群众性精神文明创建活动，推进城乡文明共建，加快社会信用体系建设，不断提高全社会文明程度。增强城乡社区卫生服务能力，巩固发展新型农村合作医疗制度，促进人人享有基本医疗卫生服务。进一步完善公共卫生服务体系，加强县乡卫生监督，开展农村医疗救助活动，提升疾病预防控制和应急救治能力，启用市新建传染病医院。落实和完善计划生育利益导向机制，加强出生人口性别比综合治理，基本实现计划生育优质服务体系全覆盖，人口自然增长率控制在7‰以内，计划生育率90%以上。加强双拥工作，提高国防教育、国防动员和优抚安置保障水平。全面完成经济普查，做好气象、档案、地方志、防震减灾、民族宗教、红十字等工作，重视发展妇女儿童、关心下一代和老龄事业。

发展民主法制，不断改善社会管理。自觉接受人大及常委会监督，主动加强与人民政协联系，及时办理人大代表、政协委员的建议和提案，认真听取各民主党派、工商联、无党派人士和人民团体意见。深入推进五五普法工作，加强基层民主法制建设，扩大村民和社区居民自治，推动法治连云港建设，促进社会公平正义。加强行政监察、审计监督，重视支持舆论和社会公众监督，着力增强政府公信力。严格生产、加工、流通、消费环节监管，整顿市场秩序，提高食品药品质量安全，让人民群众吃得放心、用得安心。继续开展重点行业领域专项执法活动，推进企业安全标准化，促进安全生产形势持续稳定。加快建立城市应急联动系统，提高预防和处置突发事件能力。推进平安创建，维护社会稳定。进一步加强科技强警建设，强化社会治安综合治理，严厉打击违法犯罪活动，提高群众安全感和社会治安满意度，万人刑事案件发案率控制在0.46%以内。建立领导干部大接访长效机制，完善大调解机制，努力解决人民内部矛盾，社会矛盾纠纷调处成功率82.5%以上。

各位代表，实现新一年经济社会发展各项目标任务，政府必须切实加强自身建设，全面提高服务水平和行政执行力。我们将进一步加快转变职能。积极稳妥启动新一轮政府机构改革，优化组织结构，理顺部门关系，强化公共服务职能，完善经济调节机制。继续精简行政审批事项，简化办事环节。加强四级便民服务网建设，整合行业服务资源，开设12345政府服务热线，打造统一便捷的公共服务平台。进一步推进依法行政。健全民主集中、专家咨询、社会公示与听证、合法性审查等制度，完善重大事项决策规则和程序。推行领导干部学法制度，强化对公务员和行政执法人员法制教育培训，努力把依法办事的要求落实到政府工作各个层面和环节。进一步提升行政效能。大力精简会议文件，大举破解发展难题，大兴调查研究之风，深入实际、深入基层、深入群众，问政于民、问需于民、问计于民。深入开展学习实践科学发展观活动，建立科学发展评价考核体系。实行行政问责制，提高公务人员履职水平。大力推进政务公开，全面实行信息公开，加快电子政务建设，推行网上审批和电子监察，增强行政权力运行的透明度。进一步加强从严治政。坚持厉行节约，反对铺张浪费。注重从制度上、源头上加强防治腐败工作，认真落实廉政建设责任制，加大对权力部门、重点行业监督力度，加强对重点专项资金、重大投资项目审计和监察，严肃整治损害群众利益和破坏投资环境行为，严厉查处违法违纪案件，保证人民政府为人民群众谋利。

各位代表，连云港的发展已经进入新的重要战略机遇期，时代赋予我们重任，人民寄予我们厚望。新的一年，困难和挑战考验着我们，责任和使命激励着我们。让我们紧密团结在以胡锦涛为总书记的党中央周围，深入贯彻党的十七大、十七届三中全会精神，在中共连云港市委的领导下，解放思想，开拓创新，奋发有为，励精图治，为把连云港国际性海滨城市建设全面推向新阶段而努力奋斗！
